# Anti-reflective nano- and micro-structures on 4H-SiC for photodiodes

**DOI:** 10.1186/1556-276X-6-236

**Published:** 2011-03-18

**Authors:** Min-Seok Kang, Sung-Jae Joo, Wook Bahng, Ji-Hoon Lee, Nam-Kyun Kim, Sang-Mo Koo

**Affiliations:** 1 School of Electronics and Information, Kwangwoon University, Seoul 139-701, Korea; 2 Korea Electrotechnology Research Institute, Power Semiconductor Research Group, Changwon 641-120, Korea

## Abstract

In this study, nano-scale honeycomb-shaped structures with anti-reflection properties were successfully formed on SiC. The surface of 4H-SiC wafer after a conventional photolithography process was etched by inductively coupled plasma. We demonstrate that the reflection characteristic of the fabricated photodiodes has significantly reduced by 55% compared with the reference devices. As a result, the optical response *I*_illumination_/*I*_dark _of the 4H-SiC photodiodes were enhanced up to 178%, which can be ascribed primarily to the improved light trapping in the proposed nano-scale texturing.

## Introduction

Up to now, silicon (Si) has been the dominant material for high-efficiency solar cells. However, Si-based devices perform well only under the limited conditions of relatively low temperatures and power ranges. Alternatively, in the research on wide-bandgap semiconductors, silicon carbide (SiC) has shown considerable potential for both high-power and optoelectronic devices [1]. SiC exhibits a wide-bandgap (3.26 eV) and superior thermal properties, which are advantageous for high-temperature applications and solar energy conversion [2]. However, polished SiC surfaces have a natural reflectivity with a strong spectral dependence. The reflectivity is inevitably high (20-40%), due to the high refractive index of *n *= 2.7-3.5 of SiC [3]. The optical losses associated with the reflectance of incident radiation are among the most important factors limiting the efficiency of a solar cell [4]. Therefore, photovoltaic cells normally require special surface structures or materials, which can reduce reflectance. A common solution is utilization of antireflection coatings based on interference, such as transparent layers of SiO_2 _and Al_2_O_3 _[5]. However, such coatings worked only in a limited spectral range, and more efficient reflection reduction in a broad spectral range has been achieved by surface texturing, which can normally be accomplished by wet or dry etching. In principle, the wet etching of SiC can be done only with molten KOH at over 500°C, which is not a practical method. For that reason, dry etching with fluorine species, such as SF_6_, and CF_4_, is considered as the desirable method to form the textured surface of SiC [6].

In this article, we report a method for forming nano-scale-textured structures on 4H-SiC surfaces so as to reduce the surface reflectance of SiC. An inductively coupled plasma (ICP) etching was employed to form the structures, and the performance of the SiC photodiode cells was compared to that of reference cells without surface nano-scale texturing.

## Experimental

**Figure 1:**
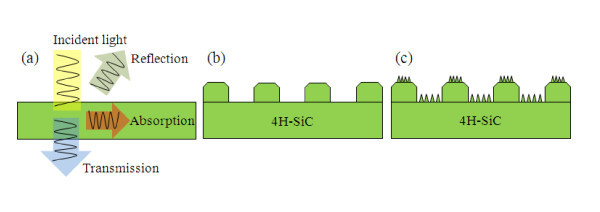
**Schematic view of the 4H-SiC with different surface structures**. **(a) **Reference cell, **(b) **micro-honeycomb structures, and **(c) **nano-honeycomb structures.

**Figure 2:**
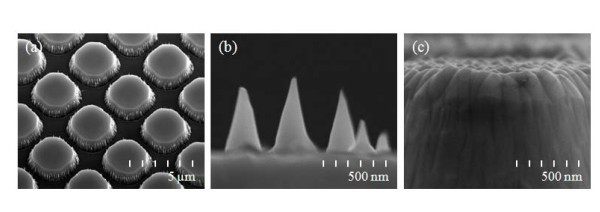
**SEM images of representative "as-manufactured" structures**. **(a) **The image shows the *nano-honeycomb structures *created by the photolithographic process. The detailed images show the rough surface on the bottom side **(b) **and the top side **(c) **of the *nano-honeycomb structures *created by the ICP-etching process using the gaseous mixture of SF_6 _+ O_2_.

Figure 1 shows the three different surface types of samples on 4H-SiC wafers that were prepared. In order to form nano-scale-textured honeycomb structures on the 4H-SiC surface, we first fabricated nano-structure patterns of the SiC surface. The samples were first cleaned in H_2_SO_4_:H_2_O_2 _= 4:1, followed by a BOE dip to remove the native oxide. The so-called nano-honeycomb etching process was performed in the following steps. First, to prepare a dry etching mask, a 100-nm Ni layer was sputtered and patterned by a conventional photolithographic process. A plasma-etching process was performed using SF_6 _plasma (15% O_2 _by flowing in a total gas load of 14 sccm) with ICP discharges at 550 W and RF chuck powers that created the dc self-bias from 117 V. The chamber pressure was 50 mTorr, and the sample was placed on the chuck that was cooled by He. Then, the remaining Ni was removed from the SiC surface by the Ni etchant (HF:H_2_O_2_:H_2_O = 1:1:8). The honeycomb structures were created with a width and spacing, both of 3 μm, and a height of 100 nm as shown in Figure 2a. This method is used for forming the honeycomb structures of SiC surfaces which are referred to hereafter as *micro-honeycomb structures *[7,8]. The substrate for SiO_2_/4H-SiC was oxidized at 1150°C in O_2 _for 5 h, and then a Si layer was deposited by electron-beam evaporation to be used as a masking layer for etching. The thicknesses of the SiO_2 _and Si layers were 100 nm and 1 μm, respectively. Nano-scale texturing was performed using SF_6 _plasma (17% O_2 _by flowing in a total gas load of 24 sccm), with an ICP discharge power and a chamber pressure of 550 W and 30 mTorr, respectively, and a RF chuck power that created dc self-biases starting from 49 V. The nano-scale textures on the honeycomb structures were made by ICP etching as shown in Figure 2b, c[9]. This method is used for forming nano-scale-textured structures of SiC surfaces, referred to hereafter as *nano-honeycomb structures*, utilized the naturally roughened SiC surface morphology when the overlying Si turns into the so-called black Si by the ICP etching. After the black Si layer was consumed completely, the morphology was transferred to the underlying SiC, resulting in a roughened SiC surface.

## Results and discussion

**Figure 3:**
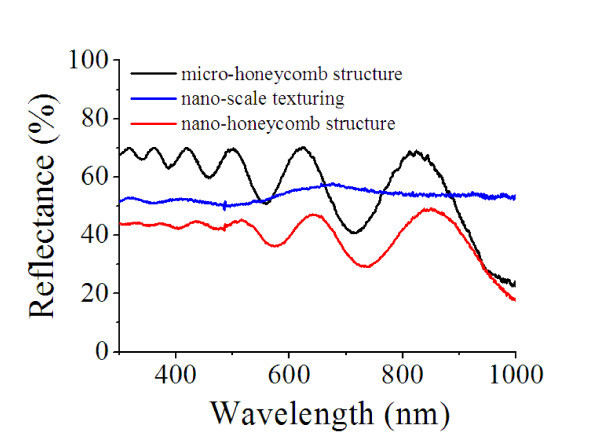
**Comparison of spectral reflectivity from 300 to 1000 nm for different surface structures**.

**Figure 4:**
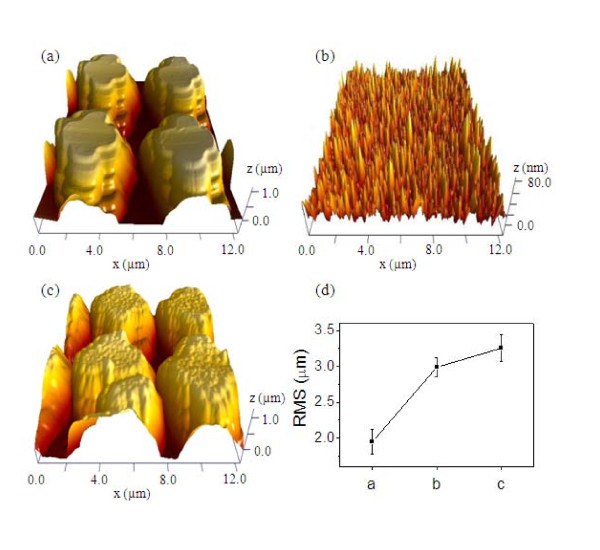
**Contact-mode AFM images of 4H-SiC with different surface structures**. **(a) **Micro-honeycomb structures, **(b) **nano-scale texturing, and **(c) **nano-honeycomb structures, as well as **(d) **RMS curve of the surface roughness.

Figure 2 shows scanning electron microscopy (SEM) images of the surface morphology of *nano-honeycomb structures*. Three different types of samples on SiC with different surface structures were examined: (a) reference structures, (b) micro-honeycomb structures, and (c) nano-honeycomb structures. The reflectance spectral dependence was studied using a UV-Vis/NIR spectrometer (AvaSpec-3648) and by AFM (N8 ARGOS) analysis. Figure 3 shows the corresponding reflectance spectra of the samples, along with those of the reference cells [10,11]. In the region of wavelengths from 300 to 1000 nm, the reflectance of the *micro-honeycomb structures *was reduced by 30% with respect to that of the reference cell. After performing the unmasked ICP etching for additional nano-scale roughening on the *micro-honeycomb structures*, the reflectance decreased by 55% with respect to the reference cell. The optical measurements of the *nano-honeycomb structures *show that the amount of absorbed light significantly increased. The decreased reflectance of the structure is ascribed to the increased roughness of the surface due to the structures formed on the surface. Figure 4 shows the surface morphology observed with an atomic force microscope (AFM) under the contact mode with a scan area of 12 × 12 μm^2^. The root mean square (RMS) of the surface roughness was calculated from the AFM images as shown in Figure 4d.

The relation between the reflectance and surface roughness can be described as [12](1)

where *p *represents the probability that, depending on the location on the rough surface, the incident photon is either absorbed with probability factor *a*, or reflected with a probability factor of *r *= 1 - *a*. As the surface roughness increases, the reflectance decreases, since more photons are absorbed. Similarly, as the RMS values of the *nano-honeycomb structures *increases, the reflectance spectral dependence decreases because of the textured surface effect on the light trapping. It can be seen from the values of reflectance for 4H-SiC with different texturing structures that the *nano-honeycomb structures *exhibit clearly improved anti-reflective properties.

**Figure 5:**
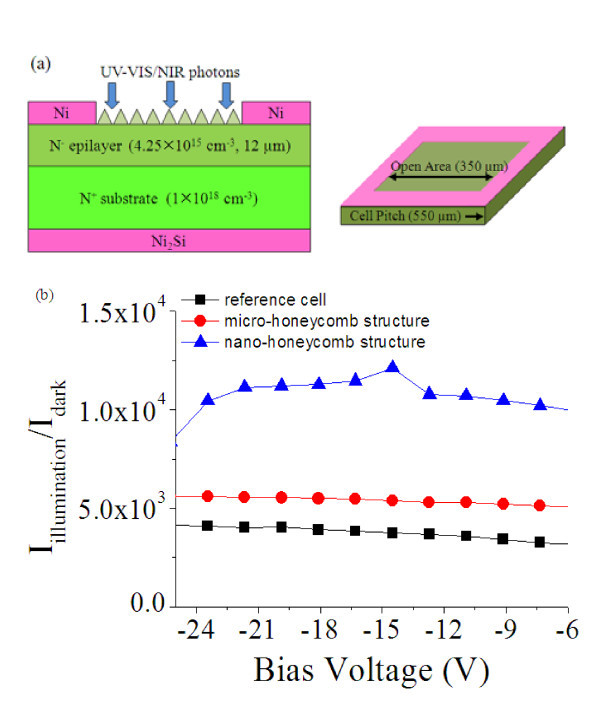
**4H-SiC photodiode structure and the optical response characteristics**. **(a) **Structure of the 4H-SiC Schottky-type photodiode with an open area of 250 × 250 μm^2^. **(b) **Optical response of the 4H-SiC photo-diodes with different surface structures.

Schottky-type ultraviolet photodiodes were fabricated on *n*-type 4H-SiC wafers with a 12-μm-thick *n*^- ^epilayer (*N_D _*= 4.25 × 10^15 ^cm^-3^) grown on *n*^+ ^substrate (*N_D _*= 10^18 ^cm^-3^) [13]. A large area ohmic contact on the back-side was formed by the sputter of a 100-nm Ni film, followed by a rapid thermal annealing process at 950°C in N_2 _for 90 s. The Schottky contacts on the front-side was fabricated by the electron-beam evaporation of a 50-nm Ni film, and a subsequent photolithographic patterning was performed to form rectangular ring patterns with widths of 550 μm and open area widths of 250 μm. Figure 5a shows the fabricated 4H-SiC Schottky photodiode structure. The open area directly exposed to radiation was estimated to be about 21% of the total device area. The current-voltage characteristics of the devices were measured by using a Keithley 4200 measuring unit. The saturated currents of the Schottky photodiodes were measured as a function of the reverse bias, both in the dark condition *I*_dark _and under UV illumination at 300 nm *I*_illumination _[14]. Figure 5b compares the optical response (*I*_illumination_/*I*_dark_) of the photodiodes measured from the *micro-honeycomb structures *and *nano-honeycomb structures*.

The photocurrent shows a slight increase in the case of the *micro-honeycomb structures*, while a significant increase in optical response can be observed in the *nano-honeycomb structures *compared with the reference cell. The comparision of the photodiode properties for different structures are summarized in Table 1. For the reference cell, the measured *I*_dark _and *I*_illumination _are 1.37 × 10^-11 ^and 5.55 × 10^-8 ^A, respectively, which results in the response of 75.4 A/W under the reverse bias of 20 V (see Table 1). The response values of 259.5 A/W at -20 V were obtained at nano-honeycomb structures, as the optical reponse is increased by 178%. The optical response values at -20 V increased by 37 and 178% for *micro-honeycomb structures *and *nano-honeycomb structures*, respectively. The increased photocurrent gain is because the surface reflectance was reduced and the amount of absorbed light was increased with the *nano-honeycomb structures*. The results suggest that we can enhance the electro-optical response of the photodiodes by the anti-reflective effect of the nano-honeycomb-textured structures.

**Table 1:** Comparison of the Schottky-type ultraviolet photodiode properties for different structures

Structure	*I*_dark _(A)	*I*_illumination _(A)	Response (A/W)
Reference cell	1.37 × 10^-11^	5.55 × 10^-8^	75.4
Micro-honeycomb	1.41 × 10^-11^	6.32 × 10^-8^	85.8
Nano-honeycomb	1.94 × 10^-11^	2.18 × 10^-7^	259.5

## Conclusions

In summary, we proposed a method for fabricating nano-scale-textured structures on 4H-SiC surfaces to reduce reflection. After a conventional photolithography process to form the *nano-honeycomb structures*, the surface of 4H-SiC wafer was etched by ICP using a SF_6 _+ O_2 _gas mixture. We demonstrated that the reflectance of the *nano-honeycomb structures *has significantly reduced by 55% compared with the reference cell. The reflectance was reduced because the roughness of the surface was increased. As a result, an optical response (*I*_illumination_/*I*_dark_) was increased by 178% for the *nano-honeycomb structures*, and an improved photocurrent was obtained from the subsequently fabricated 4H-SiC photo-diodes. The textured surface resulted in the reduction in reflectivity, which indicated that the amount of absorbed light increased because of efficient light trapping. It has been shown that the *nano-honeycomb structures *have proven as effective anti-reflective surface structures, which may open opportunities for the design of efficient photovoltaic cells on 4H-SiC.

## Competing interests

The authors declare that they have no competing interests.

## Authors' contributions

MSK and carried most of the experiments. SJJ participated in the fabrication of micro- and nano-structures and analysis. WB and JHL performed the analysis of experimental data and measurement results. MSK prepared the manuscript initially. SMK conceived of the study and participated in its design and coordination. All authors read and approved the final manuscript.
